# Social media in surgery: evolving role in research communication and beyond

**DOI:** 10.1007/s00423-021-02135-7

**Published:** 2021-02-28

**Authors:** Rebecca Grossman, Olivia Sgarbura, Julie Hallet, Kjetil Søreide

**Affiliations:** 1 Oxford Centre for Diabetes, Endocrinology and Metabolism, University of Oxford, Oxford, UK; 2 Department of Surgical Oncology, Cancer Institute of Montpellier, University of Montpellier, Montpellier, France; 3 IRCM, Institut de Recherche en Cancérologie de Montpellier, INSERM U1194, Université de Montpellier, Institut régional du Cancer de Montpellier, F-34298 Montpellier, France; 4 Department of Surgery, Odette Cancer Centre, Sunnybrook Health Sciences Centre, Toronto, Ontario Canada; 5 University of Toronto, Toronto, Ontario Canada; 6 Department of Gastrointestinal Surgery, Stavanger University Hospital, Stavanger, Norway; 7 Department of Clinical Medicine, University of Bergen, Bergen, Norway

**Keywords:** Social media, Research communication, Research dissemination, Internet, Influence, Gender diversity, Impact, Surgical research

## Abstract

**Purpose:**

To present social media (SoMe) platforms for surgeons, how these are used, with what impact, and their roles for research communication.

**Methods:**

A narrative review based on a literature search regarding social media use, of studies and findings pertaining to surgical disciplines, and the authors’ own experience.

**Results:**

Several social networking platforms for surgeons are presented to the reader. The more frequently used, i.e., Twitter, is presented with details of opportunities, specific fora for communication, presenting tips for effective use, and also some caveats to use. Details of how the surgical community evolved through the use of the hashtag #SoMe4Surgery are presented. The impact on gender diversity in surgery through important hashtags (from #ILookLikeASurgeon to #MedBikini) is discussed. Practical tips on generating tweets and use of visual abstracts are presented, with influence on post-production distribution of journal articles through “tweetorials” and “tweetchats.” Findings from seminal studies on SoMe and the impact on traditional metrics (regular citations) and alternative metrics (Altmetrics, including tweets, retweets, news outlet mentions) are presented. Some concerns on misuse and SoMe caveats are discussed.

**Conclusion:**

Over the last two decades, social media has had a huge impact on science dissemination, journal article discussions, and presentation of conference news. Immediate and real-time presentation of studies, articles, or presentations has flattened hierarchy for participation, debate, and engagement. Surgeons should learn how to use novel communication technology to advance the field and further professional and public interaction.

## Introduction

In less than two decades since its inception, social media has had a dramatic impact on science dissemination, journal article discussions, conference news, and sharing of ideas and findings [1–4]. Social media relates to all internet-based applications used for social interaction in real time, be it for personal or professional use. It has changed several aspects of how, what, when, and with whom we communicate—this holds true not only in personal life, but more so in professional aspects and academia [4–6].

Medicine is no exception and surgeons have taken to the social media platforms with varying but increasing activity [7–9]. This method of research communication requires both attention, curiosity, critique, and scientific research. With several new opportunities for research communication, knowledge dissemination, education, and debates in real time, social media platforms are important tools for surgeons. However, in addition to opportunities and advantages, there are concerns, caveats, and pitfalls to this technology as well.

In this invited review, we present some of the aspects related to social media use for surgeons and how it influences modern research communication.

## How surgeons use social media platforms

Since the invention of the printing press, methods for staying up to date with the latest medical evidence have evolved alongside the practice of evidence-based medicine itself. The speed of this evolution increased exponentially when the first scientific journals began to make their papers available online. *The BMJ* was the first major medical journal to launch a website, in 1995, and went fully online in 1998 [10]. From the mid-1990s, journals started offering the option to sign up for e-mail updates and RSS feeds (a method that allows a user to receive aggregated updates in a standardized format from multiple news sources).

Next came the *blogs* [11] (a term coined in 1997) and *podcasts* from the 2000s (the name was first used in 2004) [12]. Medical versions of these media are still incredibly popular. They allow journal editors and individual doctors to discuss, critique, and share important studies. Podcasts can be listened to at leisure, such as during commutes and exercise. Creating and maintaining them can be time-consuming. Hence, their use by surgical journals for communication with readers has been relatively inconsistent.

When Facebook (Facebook Inc., Menlo Park, CA, USA) was created in 2004, personalized news feeds became more appealing than RSS feeds [13]. Articles could be shared easily by friends and colleagues. More social networks then rapidly developed, and surgeons adopted them for socializing, networking, research, and practice promotion. Among colorectal surgeons in Australia and New Zealand, 59% reported using social media (SoMe) for networking and 9% for research purposes [14]. A summary of these platform types are found in Table 1. Among the social media tools available, some are more popular among the surgical community than others. While only used by approximately one in five US adults [15], Twitter (Twitter, Inc., San Francisco, CA, USA) has become popular among healthcare professionals worldwide, for sharing information and online content; users can use it for both broadcasting and interacting [16]. The adoption of social media by the surgical community has been gradual. Twitter seems to be one of the platforms with largest uptake by surgeons [3, 8, 17, 18].

**Table 1:** Social media platforms and use among surgeons for research dissemination

Type	Description	Examples	Examples of use
Social networks	Software that allows people to connect, either with known contacts or strangers who share similar interests via groups and hashtags, and share multimedia and links	Facebook, Instagram	Some journals have Facebook and Instagram accounts to share papers and visual abstracts, and some groups such as the International Bariatric Club hold themed online chats
Business networks	As above, with a focus on sharing professional information and resumes	LinkedIn	Share professional updates and blog posts, participate in group conversations
Science and research networks	Allow researchers to log and share publications and other academic work	ResearchGate, Mendeley	Log and share papers, request access to papers that are behind a paywall
Blogging	A way to publish articles online in various formats in a user-controlled way	WordPress, Tumblr	Editorialize and summarize work in plain language. Useful for public engagement
Microblogging	Character-limited posts, able to share multimedia and URL links	Twitter	Used by large number of journals and researchers to share research summaries, links to papers, and teaching cases
Video sharing	Allow users to “vlog” and share videos to followers or the general public	YouTube, AIS (Advances in Surgery), WebSurg	Used for sharing educational videos, lectures, and videos of surgical techniques
Gaming networks	Allow players to play together, stream games, and chat online	Twitch	Potential for simulation training
Wiki-based	Collaborative editing of an information source	Wikipedia, SAGES Surgical Wiki	Can contribute to evidence-based surgical knowledge, citing scientific papers as sources
Group-messaging service	Allows users to send instant messages across different platforms to contacts and channels	WhatsApp, Signal, Telegram, WeChat	Can share papers with contacts
Discussion fora	Website that allows social news aggregation, content rating, and discussion	Discord, Reddit	Ability to chat online about new papers

### Social networks

Facebook is one of the most used platforms; e.g., in the USA, it is used by over two-thirds of the adult population [15]. Despite this, it is infrequently used by individual surgeons for purely professional purposes, as it encourages users to share personal information such as family members and tagged pictures of private events that are not always appropriate for professional interactions. However, as it allows a “closed” membership, it is used by some societies to facilitate discussions and engagement [19]. This approach leverages members’ existing social media accounts and familiarity with the platform to engage them in case discussion and webinars, thereby broadening participation compared to society-specific web platform that requires separate processes and login. It can also add a socializing and networking aspect to discussions that was previously achieved mostly at in-person conferences. Instagram (Facebook Inc., Menlo Park, CA, USA) is owned by Facebook and is used by a younger demographic. Due to it being primarily an image-sharing platform, it is popular within plastic and aesthetic surgery [20, 21]. It is also used by private medical institutions to advertise medical activity and facilities [17].

### Blogs

Surgical blogs have experienced a recent renaissance [22, 23]. Blogging is a good vehicle for topics not covered in traditional scientific literature and that require a more extensive coverage than the short format proposed on the other platforms. Surgical blogs may be targeted to a specific audience, such as the Association of Women Surgeons (AWS) blog [23]. This example generates high numbers of unique user views and provides unique content, such as topics including “graduate and postgraduate education” and “family life,” among others. Blogs can also be used by scientific journals. *BJS* created a blog that allows the journal’s editors and authors to post articles on themes that are not traditionally found in a printed journal (The Cutting Edge blog https://cuttingedgeblog.com). These posts stimulate debate and allow reflection in an alternative way. In addition, they allow the communication of the findings of surgical research in plain language, to make it available to the general public. This is an important method of countering widespread medical misinformation online. Surgical blogs can thus be used for knowledge dissemination and translation, as well as patient engagement in research. Research outputs can be shared with the surgical community, policy-makers, and patients in formats more accessible than scientific articles. Clinical trials and ongoing studies can be shared to improve centers’ participation and patient recruitment [24]. Interactive features can also be used to obtain input and feedback through the design, conduct, and interpretation of research projects to foster patient and service user engagement that is key to impactful research [25].

### Video sharing

YouTube (Google LLC, San Bruno, CA, USA) is the most popular form of social media in the USA, with three-quarters of adults using the platform [15]. It is particularly useful for sharing educational videos, recorded lectures, webinars, and instructional procedure videos. Research suggests there is broad uptake of these videos [26, 27]. Several surgical specialties have used this form of knowledge distribution for various procedures [28–31]; however a recurring concern is the validity and usefulness of the content posted [32–34]. There is no surgical content curator on YouTube. Hence, the inexperienced surgeon may be led astray into poorly crafted and potentially erroneous videos, which is a pitfall to the widespread dissemination available by these platforms. Compared with traditional journal articles in a professional surgical journal, social platform content is not vetted to a given standard with editorial input or external referees. Thus, bias (unconscious and conscious) and errors are rife. In a cross-sectional study of YouTube videos demonstrating laparoscopic fundoplication, only 39.4% were evaluated as “good”; good-rated videos correlated with longer duration [35]. Similarly, an analysis of YouTube videos presenting D2 lymphadenectomy for gastric cancer highlighted the high variability in the quality of the technique presented using validated scoring tools [36]. Higher quality videos have supplementary commentary [26]. Trainees may also evaluate the quality of a surgical video differently to senior surgeons [34] and should therefore be steered towards the higher quality videos by their instructors.

Formal curation is provided by other video platforms, such as WebSurg and the AIS (Advances in Surgery) Channel. As with other online streaming platforms, these tools gained more visibility during the Covid-19 pandemic, when in-person congresses were cancelled and partly replaced with virtual events [37]. It is unclear what the role of these formats will be once in-person conferences resume, but their persistence seems certain.

## How it started: #SoMe4Surgery and related hashtags

**Fig. 1:**
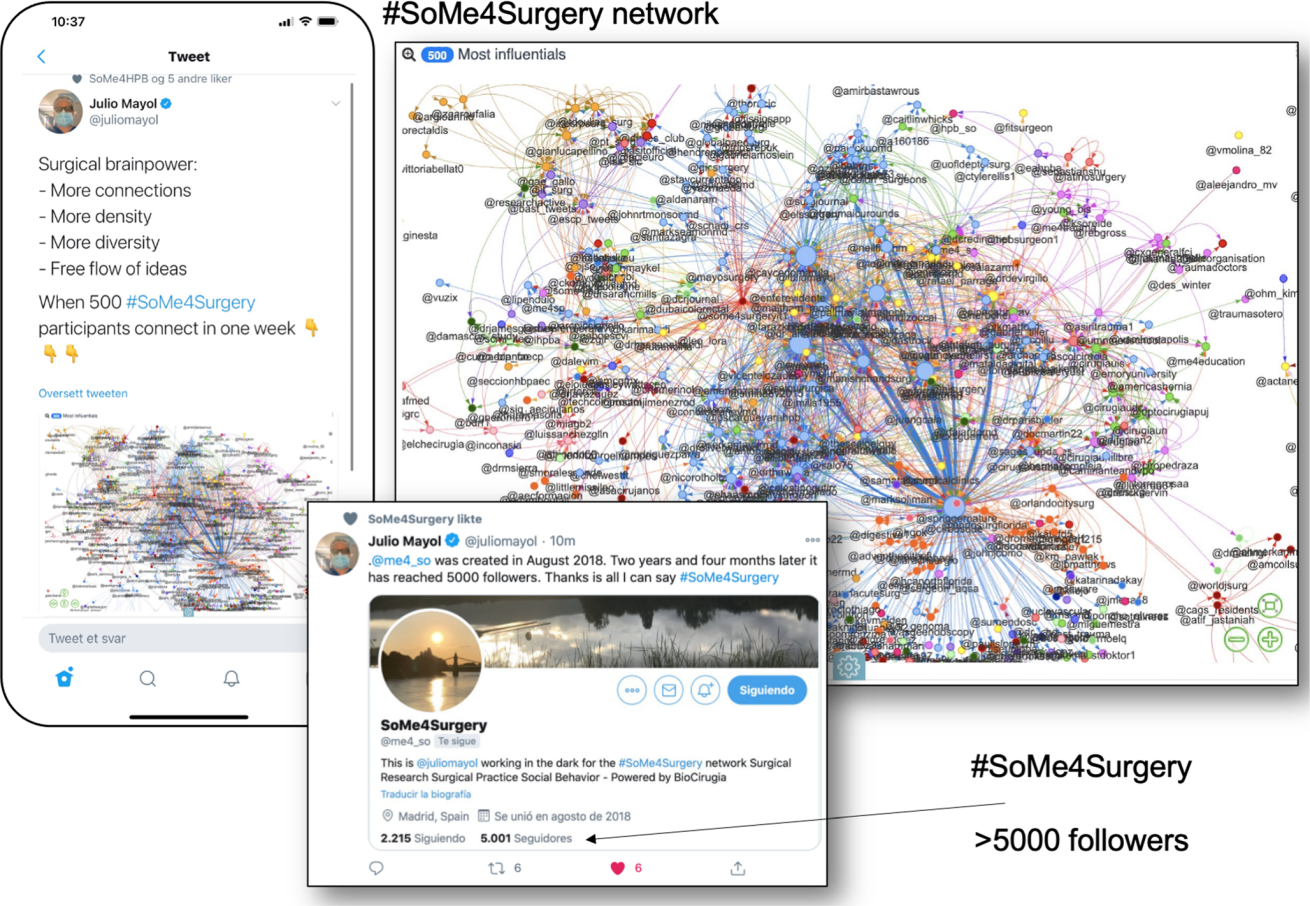
The “SoMe4Surgery” network illustrated. Created in August 2018 by Professor Julio Mayol. After 2 years and 4 months, the #Some4Surgery network and the Twitter handle (@me4_so) had reached over 5000 followers, with considerable interaction

One of the ways to maximize the benefits of social media use is to employ hashtags, which are metadata tags that are user-generated/“bottom-up” [38]. This allows users to engage in conversations that are related to a certain topic. Various surgical hashtags exist. #SoMe4Surgery is both one of the most widespread and most surgeon-specific on Twitter [39] (Fig. 1). The hashtag was developed through several phases and described in detail elsewhere [39]. Briefly, an inception phase was initiated for a connection between participants: users were actively invited to participate. Second, a dissemination phase was launched to help the spread (contagion) and the material going viral. In this phase, several tweetchats were designed, scheduled, and run. Further, a third step was the adherence phase (feedback): Twitonomy and NodeXL summaries were regularly posted on Twitter to gauge and inform activity and response. Eventually, an impact phase in which outcome was the focused gain [1, 39]. Created in August 2018, after 2 years and 4 months, the #SoMe4Surgery network and the twitter handle (@me4_so) had reached over 5000 followers, with considerable interaction (Fig. 1). Currently, a long list of twitter handles related to surgeons’ interest is available (Table 2).

**Table 2:** Social media handles for specific surgical interest groups evolved since 2017

Name	Twitter handle	Purpose	Launched
SoMe4Transplant	@SoMeTransplant	@SoMeTransplant connecting all #healthcare workers, #students and #patients interested in #Transplantation #SoMe4Transplant	September 2017
SoMe4Surgery	@me4_so	This is @juliomayol working in the dark for the #SoMe4Surgery network Surgical Research Surgical Practice Social Behavior - Powered by BioCirugia	August 2018
UroSoMe	@so_uro	We’re an international group of urologists and trainees who are interested in the utility of Social Media in Urology. Start by using the hashtag #UroSoMe !	August 2018
SoMe4Endocrine	@SEndocrine	Connecting the endocrine community	January 2019
SoMe4MITherapy	@me4_mi	Devoted to share information and to connect all those people interested in all forms of interventional therapy - Powered by Biocirugia	January 2019
SoMe4Precision	@PrecisionSo	The #SoMe4Precision community aims to identify and alleviate factors preventing the widespread implementation of personalised medicine in healthcare	January 2019
@SoMe4SurgeryPrehab	@SoMe4SurgeryPr1	Interés por el bienestar de los pacientes. Luchando por implantar los cuidados preoperatorios en las cirugías mayores	January 2019
SoMe4Peritoneum	@SPeritoneum	Peritoneal Surface Malignancies SoMe global community for all surgeons, researchers and patients... peritoneal carcinomatosis, HIPEC, PIPAC	January 2019
SoMe4Bariatrics	@BariatricsSo	Using #SoMe to fight the global obesity epidemic -#SoMe4Surgery	January 2019
SoMe4Trainees	@SoTrainees	Connecting surgical trainees from all over the world - led by @CarlosSaez_ and @juliomayol	January 2019
SoMe4IBD	@some4ibd	#SoMe4IBD connecting gastroenterologists, surgeons & patients interested in IBD Fueled by @LuisSanchezGlln & @GianlucaPellino under the auspices of @juliomayol	February 2019
SoMe4retina	@me4retina	This is @draPilarCalvo trying to help people see. Retina is our passion: clinic, teaching and research #some4retina	February 2019
SoMe4HPB	@hpb_so	HPB surgery in the twittsphere #SoMe4HPB	February 2019
#SoMe4Anesthesia	@some4anesthesia	#SoMe4Anesthesia	March 2019
Some4MDT	@MdtSome4	Multidisciplinary Team approach for coordinated care, patient at the heart of all we do!	May 2019
SoMe4Trauma	@Me4Trauma	#SoMe4Trauma is a #SoMe4Surgery initiative that aims to bring Trauma Surgery into focus on social media. @me4_so @a160186 @juliomayol Instagram: some4trauma	July 2019
#SoMe4PedSurg	@Me4Ped	A global initiative for all Surgical&Paediatric Information on #SocialMedia for Pediatric Surgeons mainly Just share it #SoMe4PedSurg-#SoMe4Surgery Movement-	July 2019
SoMe4endoscopy	@SoMe4endoscopy	#SoMe4endoscopy connecting gastroenterologists and surgeons interested in endoscopy. A #SoMe4Surgery initiative. @drfrancescopata @stevenbollipo @RashidLui	July 2019
@SoMe4MV	@some4mv	Multi-professional profile focused on mechanical ventilation and respiratory diseases in critical patient. Managed by @ventilacionmeca and @Nopanaden #SoMe4MV	July 2019
SoMe4GynOnc	@GynMe4	Social media platform promoting the goal of curing Gynecologic Cancer; hosted by @GreggNelsonERAS	July 2019
SoMe4Breast	@BreastMe4	We are #GlobalSurgeons studying and fighting with our knowledge and Daily practice to BREAST CANCER and other diseases related—-@martajimenez135	July 2019
SoMe4SurgicalEducation	This is an account for surgical education	This is an account for surgical education	July 2019
SoMe4CT	@SoMe4CT	Social Media #SoMe for the Cardiothoracic Surgery community #SoMe4CT : An attempt to unify everybody involved in the chest diseases. Powered by @jiseav	July 2019
SoMe4AmbSurg	@AmbMe4	Global iniciative [sic] for people, students, nurses, doctors and all Team related Ambulatory Surgery, #SoMe4AmbSurg , patient safety, surgery, innovation, research	July 2019
SoMe4SurgicalAI	@Me4Ai	Part of #SoMe4Surgery initiative. Platform for healthcare professionals an AI/data scientists to meet, debate, collaborate. #SoMe4SurgAI. Hosted by @HansL16	August 2019
TUGS	@T4UGIS	The Upper Gastrointestinal Surgeons (TUGS) represents all of Upper Gastrointestinal Surgery including its various subspecialties globally. #TUGS	October 2020

## Social media and social change

**Fig. 2:**
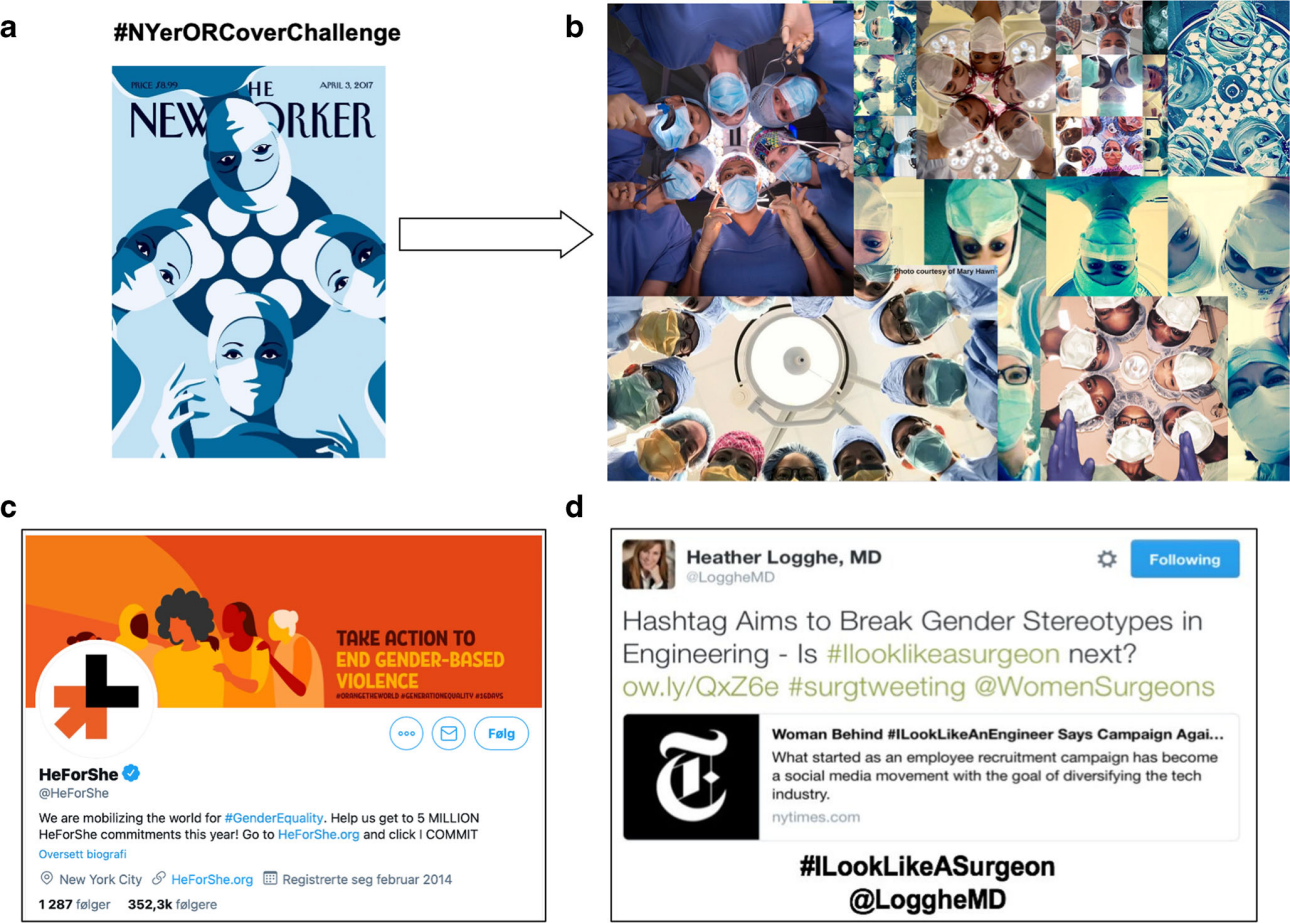
Social media and social change in gender diversity in surgery. After publication of the cover on the magazine, The New Yorker (**A**), the Twitter platform was used as a vehicle to promote women surgeons (**B**) working in institutions across the world, reproducing the cover art and thus giving a face to the thousands of women surgeons in current surgical practice. (**C**) The HeForShe Twitter handle mobilizing for gender equity. (**D**) The initial tweet by Heather Logghe starting the *#ILookLikeASurgeon* hashtag that eventually went viral

It is possible for content to be shared online in such a way that it spreads extremely rapidly, i.e., “going viral.” Examples of hashtags that went viral in the “Twittersphere” (Twitter ecosystem) and have had a huge social influence on surgery include *#ILookLikeASurgeon*, the *#NYerORCoverChallenge* (Fig. 2), and *#HerTimeIsNow*. These hashtags have celebrated diversity, raised awareness of discrimination and microaggression, and promoted the recognition of women surgeons [3]. Indeed, social media has addressed sexism in science in a wider perspective [40], stirring debate and encouraging progress towards gender equity and opportunities. As such, *#ILookLikeASurgeon* has become a global phenomenon [41] with several specialties taking up the challenge [42].

Social media allows for wider inclusion. It is now easier for junior surgeons or underrepresented groups to create a voice for themselves and make their expertise and contributions known. This helps to make them more accessible for opportunities such as invitations for speaking engagements, whereas without social media, this would be contingent on contacts and networking at meetings, which is not possible nor intuitive for all. SoMe also allows more opportunities to amplify others, support, and promote one another. This has certainly been the case for women within the American Hepatobiliary Pancreatic Association (AHPBA) with the hashtag *#hpbheroines*. While not all hashtags may go “viral,” they may become impactful, even powerful, tools in certain communities. Indeed, speaker representation of women in the IHPBA conference held in Brisbane 2020 was altered after the Twitter community noted the lack of women invited speakers. Hence, social media may bring a “diversity bonus” by fostering teams that would not have existed otherwise—be it for conferences, webinars, or research collaborations.

However, there are still limitations. Social media does not entirely protect for disparities, and geographical disparities in particular have already been identified in the use of social platforms. Independently of their utility, some platforms are preferred by users based on their local popularity [15, 43, 44]. Although access to the global conversation in surgery on Twitter is free of charge, and experts are present and active, some countries still remain underrepresented, as it has already been shown at a more global level [45]. Potential reasons for geographical disparities range from poverty and lack of resources, preventing access to the internet, to political (in countries with restricted freedom of speech), linguistical, and cultural.

## Social media engagement in research—from idea to project

It is possible for a research paper to be entirely conceived, constructed, contributed to, and disseminated via social media. The phenomenal advantage of this concept is the ability to collaborate with esteemed international colleagues with which it would otherwise be impossible to network. In addition, social media acts to “flatten the hierarchy,” allowing junior researchers to approach legends of their specialties with their own ideas, in return stimulating their own interest in research (Table 3).

**Table 3:** Social media involvement leading to new research ideas

Phase/stage	Description	Examples
Idea	Ideas can arise from online dialogues, tweetchats, commentaries, or posted articles	The idea for the paper “The Way to Man’s Heart Is through the Stomach” [46], and the collaboration between colleagues at different institutions, arose from a Twitter conversation. Twitter accounts involved in the original conversation were mentioned in the acknowledgements.
Working groups	Affinities, similar position, spontaneous organization around an idea in a tweet chat	Surgical ergonomics working group spearheaded by @GeetaLalMD following discussions on this topic on live tweets from surgical conferences
Collaborations	Collaborative studies either prospective or retrospective	Prospective: LiverGroup.org; PancreasGroup.org Surveys Retrospective COVIDSurg collaborative [47–51]
Recruitment	Post adverts to recruit sites and patients to studies, and post updates on how recruitment is progressing [52]	Sunflower study
Application	Tweet surveys have known a remarkable popularity during the Covid-19 pandemic	Several Covid-19 surveys [53, 54]
Journal selection	Different policies on Twitter	Ex. *BJS*—since 2014
Dissemination	Conference live-tweets, online congresses	Surgical conferences with monitored Twitter activity [55–59]
Innovative formats	Specific interactions around ideas or research limited to the use of a SoMe platform	#HPBdailyRead, shared HPB-specific info on a hashtag for followers

## Post-production

Following publication, there are numerous methods of disseminating research to maximize impact. Some advice for promoting papers on Twitter are found in Table 4.

**Table 4:** Advice for promoting papers on Twitter

Annotate: give different information than just the title, explain the study	
Remember to post the URL to the study and ensure no broken links	
Ensure abstract is available to read	
Use medical hashtags	
Use images, emoticons, GIFs, or visual abstracts	
Tag the research team (can be tagged on a posted photo if characters limited)	
Tag key Twitter users that may be interested in the study: surgeons, patients organizations, surgical associations, policy-makers (locally or internationally)	
Ask questions about the implications of the work—open-ended or use Twitter polls	
Like, thank, and reply if other users RT the study or comment on the study	
Follow Altmetric reports if available—will show tweets and social media posts that one may have missed otherwise	

### Tweetorials

**Fig. 3:**
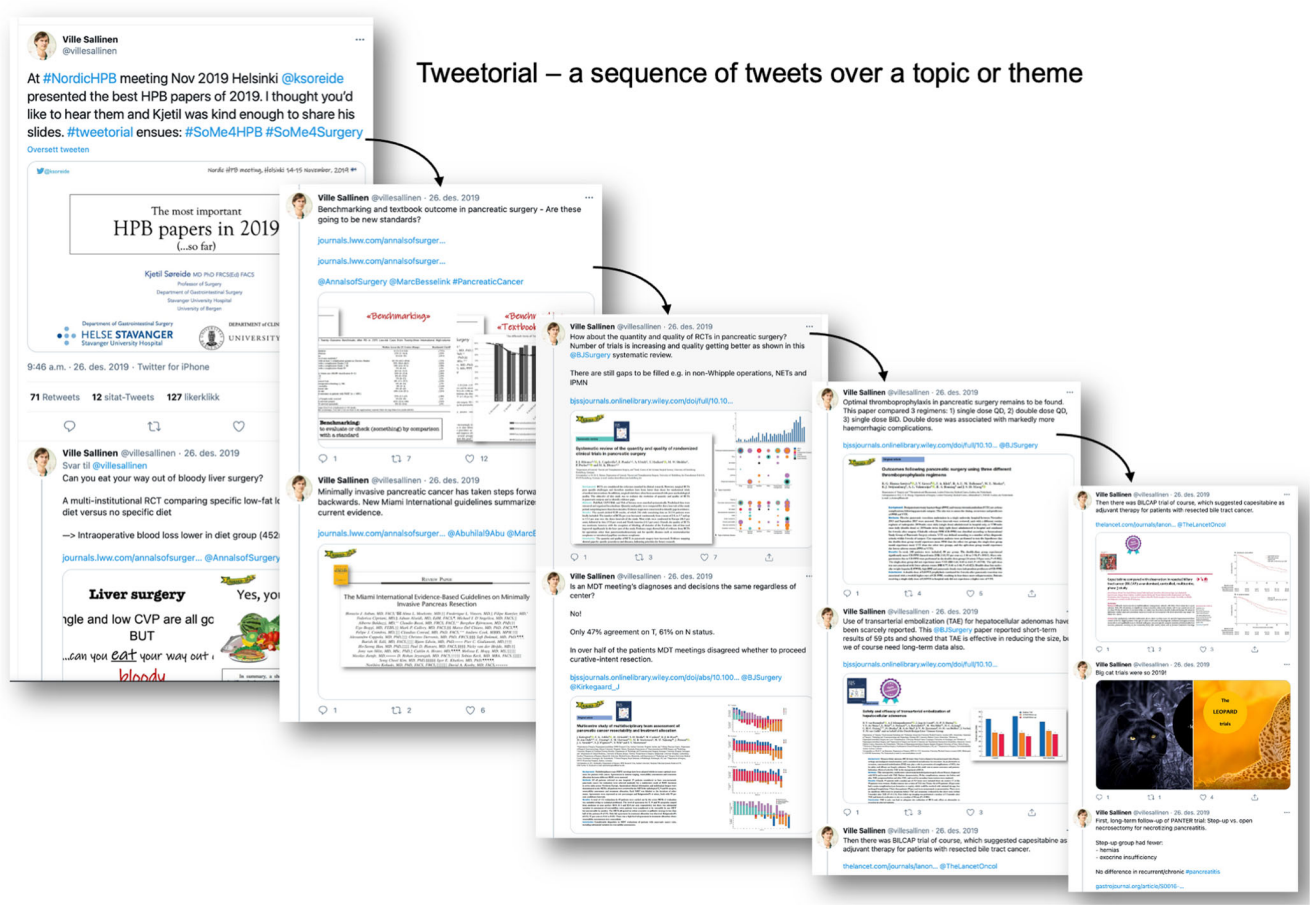
Example of a tweetorial. After the 2019 Nordic HPB meeting in Helsinki, Finland, a tweetorial was presented by the host Ville Sallinen of a summary presentation. The full tweetorial can be read at https://twitter.com/villesallinen/status/1210119626165755905. The use of social media allows non-participants to take part and engage in content, allowing for a wider distribution and input to a meeting

Tweetorials, or tweet tutorials, are explanatory Twitter threads posted on an academic topic [60] (see example Fig. 3). They are often posted by the authors of papers to explain their findings in plain language and employ the use of images from the paper as well as animated GIFs to illustrate the topic. Because the paper is explained in short, 280-character snippets, they are effective tools to summarize the key points of a paper. The caveat is that if threads are too long, the audience may not read to the end (a reflection of the short attention span encouraged by the use of microblogging sites).

### Tweetchats

A tweetchat is an open conversation on Twitter, usually based around the use of a hashtag, and often guided by one or two accounts that may post a series of questions designed to stimulate discussion [16]. They are employed by medical journals, researchers, and journal clubs to promote published papers and are highly effective in increasing the alternative metrics (Altmetrics) and reach of the paper, with impressions (number of times the hashtag has been viewed) sometimes in the millions. In one study [16], individual tweets from a journal tweetchat were extracted using Twitter analytics in addition to third-party applications NodeXL and Twitonomy, which showed that 37 Twitter accounts posted 248 tweets or replies, with only 58.5% identified using the hashtag of the tweetchat. It is therefore possible for the conversation to quickly become disorganized and difficult to follow, particularly if participants forget to include the hashtag. Third-party applications may also overestimate the reach of hashtags.

### Condensed communication—from 280 characters to visual abstracts

**Fig. 4:**
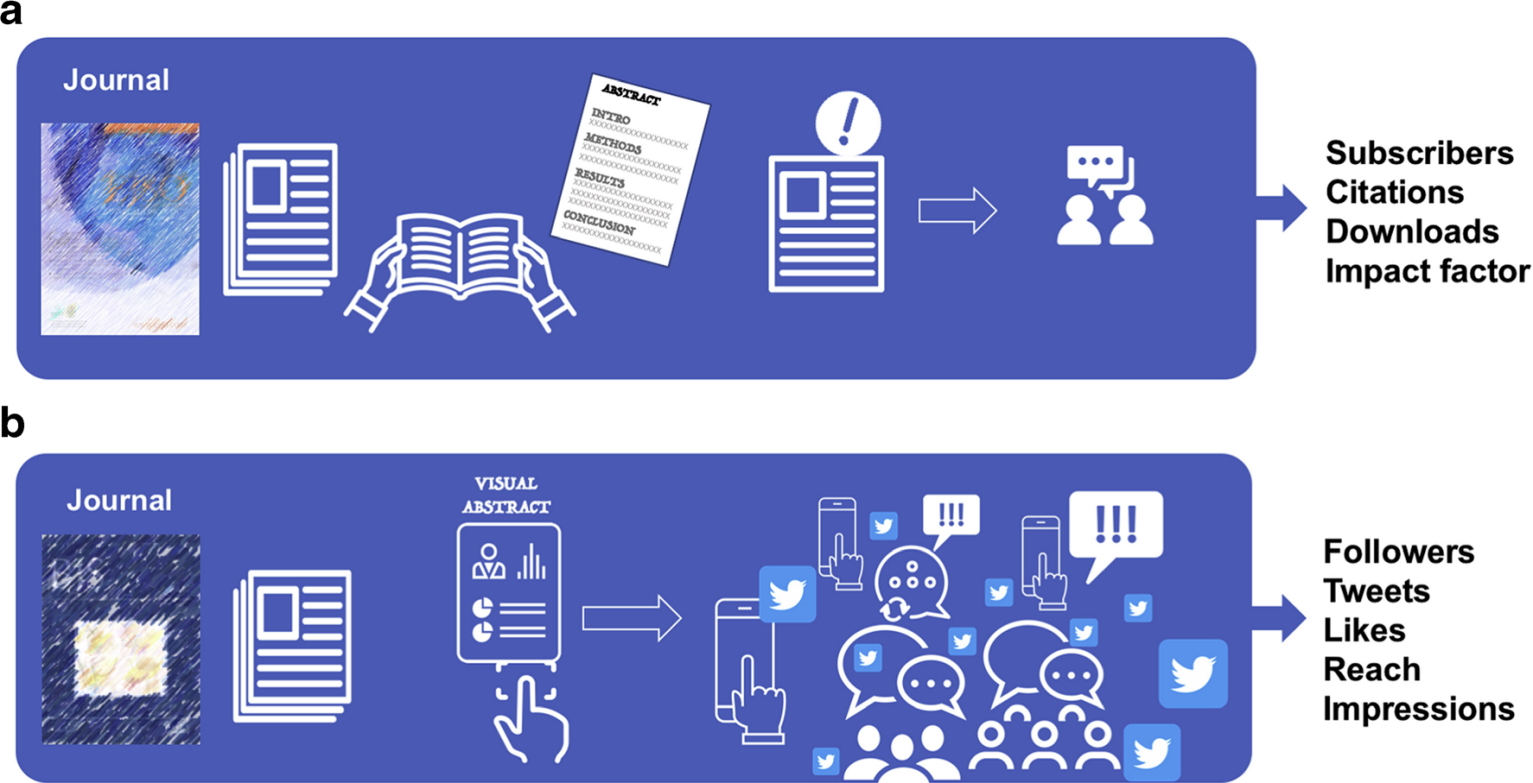
Traditional and alternative metrics for measuring research output. The metrics used to evaluate the impact of social media activity are changing; in **a**, the traditional focus on citations and impact factor (IF; Journal Citation Reports ©Clarivate Analytics) is increasingly challenged by **b** alternative metrics, dubbed “altmetrics” for short (collected by various projects, including Altmetric, Plum Analytics, and ImpactStory), that collect views and mentions over a wide range of sources, including (but not limited to) peer reviews on faculty of 1000, citations on Wikipedia and in public policy documents, discussions on research blogs, mainstream media coverage, bookmarks on reference managers like Mendeley, and social networks such as Twitter. Reproduced with permission from Elsevier under the Creative Commons license from Søreide [3]

The traditional form of condensed presentation is through the scientific abstract—either as a meeting abstract or as a condensed part of a journal article (Fig. 4). The structured part of an article usually consists of about 250 words following the IMRaD (introduction, methods, results, and discussion) structure [61].

**Fig. 5:**
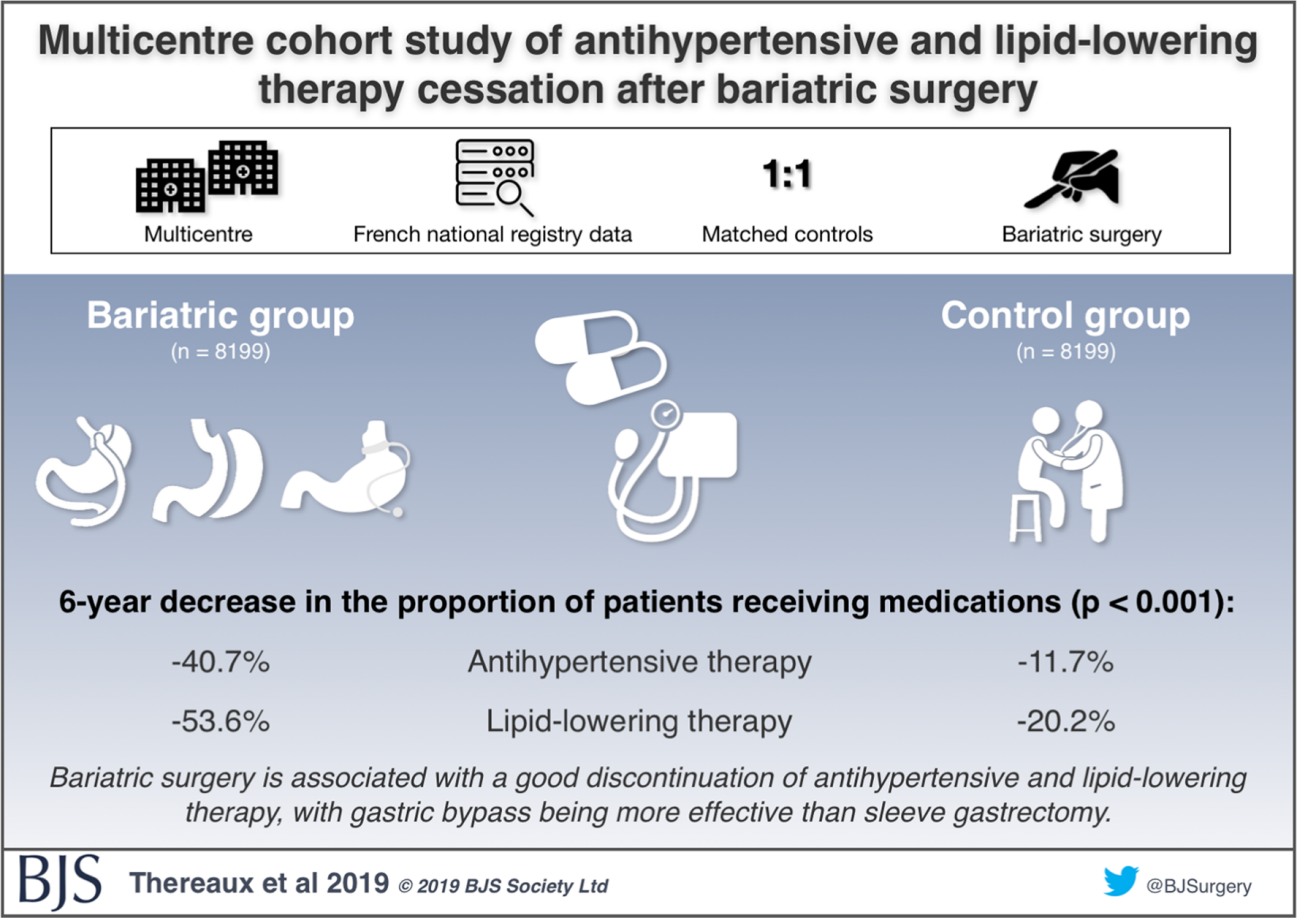
The visual abstract. An example of a visual abstract used to post summary content of a study published in *BJS* (courtesy of R. G.). The tweet with the visual abstract also contains a few notes on the study and should also contain a link or shortcut to the paper itself, hence acting as a teaser to attract the reader to study the full-text version. Thus, the purpose of the visual abstract is to be simplistic in message, yet will not replace the need to read and understand the full depth of the paper

The “visual abstract” has not replaced but added visual value to the traditional scientific abstract [62–64]. Most surgical journals now post visual abstracts of many of their published studies (Fig. 5). Indeed, some journals, like the *Journal of American College of Surgeons* (*JACS*) and *JAMA Surgery*, require authors to provide a visual abstract of their work at the time of article submission or revision. Suggested information to include in a visual abstract are found in Table 5 [65].

**Table 5:** Standard or essential information to include in a visual abstract

Guideline	Rationale
State the question or purpose of study	Place the study in context
Describe research design	Make quality of evidence clear
Report primary outcome	Reduce reporting bias
Report *p*-value or outcome measure	Allow reader interpretation
Label citation of the article and provide full URL link to article	Facilitate easy access to main source; contribute to Altmetric score
Use same language as in article	Consistency, reduce bias
Use images with permissions to reuse	Prevent violation of copyrights
External review (by someone unfamiliar with the work)	Obtain feedback on readability, ensure credibility, and identify bias
Post on social media platforms	Allow for dissemination and engagement

Modified and developed from Ibrahim [39]

## Specialized feeds for disseminating research

Social media platforms such as Twitter are immensely useful for keeping up to date with the latest research. However, feeds can often be cluttered by a mixture of useful links to journals, distracting viral videos, and photographs of pets. A relatively new trend is of Twitter accounts that act to filter out the noise (Table 6). These are usually run by volunteers with a passion for academic medicine, who act to curate and filter the papers by specialty, as opposed to the personal accounts of doctors and scientists, who may post mainly their own work. Some are run by bots. Following the creation of the #SoMe4Surgery hashtag and @me4_so (SoMe4Surgery) Twitter account [39], numerous subspecialty surgical accounts were created to curate content, with some examples shown in Table 2.

**Table 6:** Examples of Twitter accounts that curate surgical content

Twitter name	Twitter handle	Description	Joined
Transplant Library	@Transplant_TL	Transplant Library: the world’s most used online information resource for transplant professionals. One Place, All Evidence!	March 2013
Surgical Research	@SurgeryScience	Latest surgical research journal papers as they’re published | Academic updates and CME | @DrEdFitzgerald	August 2014
Colorectalsurgery	@colorectaltweet	Developments and Research in coloproctology - harvests the best tweets from research on #colorectalsurgery hashtag and from the colorectal twitterazzi	May 2016
#ColorectalResearch	@colo_research	A virtual journal of the best research from #colorectalresearch - jointly authored from @bjsurgery @colorectaldis @techcoloproctol @dcrjournal	July 2017
Surgery Highlights	@SurgeryGo	For #Surgeons around [globe emoji] to stay engaged with #Basic & #Clinical #Surgery, #Surgical #Innovation & #Research . originals in Favorites	July 2017
Roux Library	@UpperGIResearch	Virtual Twitter library curating the latest upper gastrointestinal surgery research. #UpperGIResearch #HPB #Bariatrics #OG #FOAMed #EBM #SurgicalResearch	July 2017

## Return on investment: does social media add to science communication?

Network science is an emerging and evolving specialty. Recent research suggests that sharing a traditional journal article on social media increases attention and downloads [66]. This can be facilitated in many ways, including discussing the study in a blog [66], posting short tweets, or sharing visual abstracts, images that summarize the main findings in a research paper [63–65, 67]. The latter has shown to increase attention to the study results in an appealing way with increased distribution and downloads as a result [62].

The importance of social media has thus become such that it is viewed as standard to disseminate articles via SoMe platforms [68]. SoMe is an increasing area of investigation across several surgical disciplines, with current studies pointing to differences in activity, with both positive and unchanged effects across various disciplines and outcomes [3, 6, 69–74]. Some journals, such as *BJS*, even summarize surgical Twitter activity from 1 month to another, to show highlights of debates and opinions [75–78].

Furthermore, social media communication of research results can reach broader audiences that traditionally have difficult access to scientific communication owing to journal paywalls and hermetic language. For instance, patient advocacy organizations and policy-makers can be kept appraised of new research findings in real time. This contributes to relaying and democratizing information for patients as well as impact on care processes and policies.

## New metrics of impact: Altmetrics and more

**Fig. 6:**
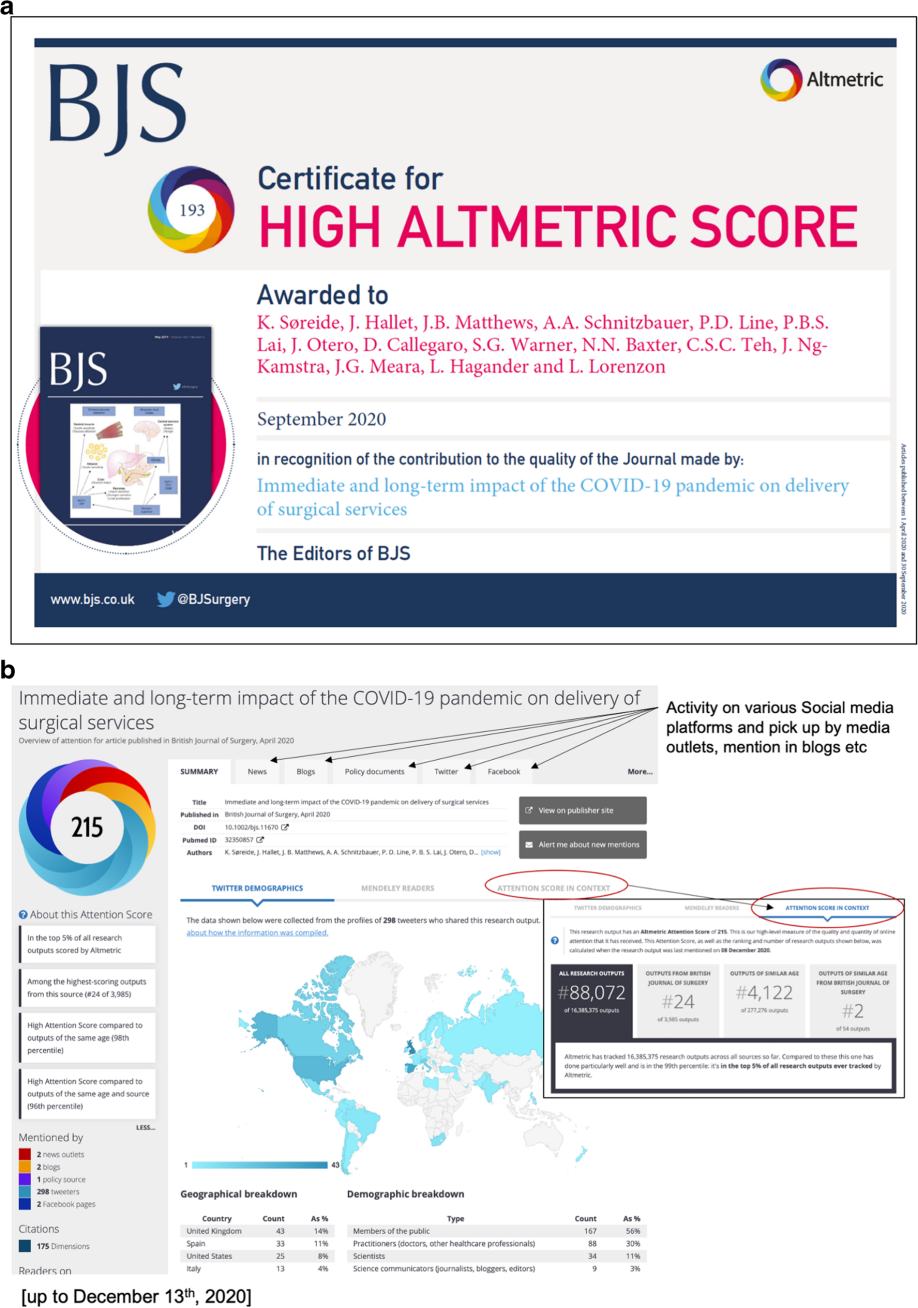
An example of an Altmetric certificate. Some journals are now providing certificate to high Altmetric scoring papers, as shown in an example (**a**). The Altmetric score captures activity on social media platforms and news outlets, and gives an impression of immediate attention and global reach (**b**) but it is unclear to what degree this returns on citations or actual impact

For what it is worth and with all its flaws, scientific papers and researchers have traditionally been evaluated by the impact of their work based on where they publish (rank of journal and its impact factor), how many times their work is cited (for any given paper and time period), and their accumulating *H*-index (the sum of citations accrued over years of active research) [79, 80]. With the emergence of social media platforms, new bibliometric profiles measuring impact and exposure of scientific research online have been introduced as an Altmetrics (Fig. 6) to traditional bibliometric outcomes. Indeed, the influence of social media activity has become an interest for academics for several reasons [46], not only to gauge the actual tweets and retweets, but also whether this activity may turn into higher citation rates and wider impact of the research [47–49]. Currently, very few studies exist in general surgery, but one study found a positive correlation between Altmetric scores and citations [74]. Notably, Altmetric scores should not necessarily be used as a surrogate marker for evaluating research performance, impact, or exposure. It is possible, however, that as the use of social media for distributing and sharing scientific research continues to expand, that exposure on such platforms could impact future interest or studies.

One study found that Twitter activity was associated with higher research citation index among academic thoracic surgeons [50]. In a unique randomized study conducted among a network of cardiothoracic surgical journals [51] called the Thoracic Surgery Social Media Network [52, 53], the investigators compared tweeted articles to non-tweeted articles for their citation output at 1-year follow-up. When compared to control articles, tweeted articles achieved significantly higher Altmetric scores, with more citations at 1 year, highlighting the durable scholarly impact of social media activity [2]. Multivariable analysis showed that independent predictors of citations were randomization to tweeting, higher Altmetric score, open-access status, and exposure to a larger number of Twitter followers as quantified by impressions [51]. This is consistent with previous social media research [52].

Based on data from subspecialty surgery, as mentioned above for thoracic surgery [51] and also for vascular surgery [54], there seems to be positive correlation for some papers with higher citations after social media exposure. However, such correlation was not found in two studies on plastic and aesthetic surgery papers [48, 70] and only weak associations were found for Covid-19 papers [55]. Hence, it remains to be shown if these alternative metrics related to tweets, mentions in blogs, displays in news outlets, etc. will have any impact on the majority of research being published in surgical journals.

## Some concerns of SoMe

Social media is an unedited, live, non-curated, short text–based forum where statements, claims, and voices may go unchecked yet widely distributed. There is a risk for false claims and so-called fake news [56] to appear—even in the scientific literature. Followers, readers, and participants need to keep a critical mind when reading and retweeting content—this has become even more prudent and scrutinized after the 2020 US presidential election, with impact of social media usage beyond political campaigns.

Creation of echo chambers is a real risk with SoMe, with a huge number of followers applauding a statement or claim, while shooting down any counterarguments (real or not). Sound debates really are fruitful, but “trolling” and “bullying” are threats to the sound debates in several instances.

There is a risk for influencers being heard and having a voice based on a strong presence and wide activity on social media, rather than being true experts or contributors to the scientific field or community per se. The discrepancy between the SoMe presence and the actual contribution to the field has been dubbed the “Kardashian index” by Hall [57]—simply “being famous for being famous,” without really having contributed. Identification of “influencers and Twitter stars” may be largely variable between specialties and countries [58, 59, 81, 82]. Few investigations exist into the matter, but some studies [82, 83] from the field of cardiology found that expert scientists have significantly more relevant audiences than so-called twitteratis (i.e., persons being influential through very high activity on Twitter) while the need for active participation and implication from the forefront of academia is still high. In addition, as long as true equity in academia remains a utopian dream, having a high Kardashian index may not be a bad thing, if it allows the playing field to be leveled; having fewer publications and citations does not make one’s opinions less valid, nor their contributions less valuable. As in any field of debate, it is crucial to separate actual information from mere noise.

Language and participation restrictions should also be noted. SoMe activity and use of various platforms are widely variable across the world. This can lead to the point of geographic exclusion as some regions are not participating or largely underrepresented in debates or discussions that become “truth” or “representative” of an opinion. Also, the use of professional and private use is very different between professionals [84], with some mixing both, others strictly professional and some merely for fun or social networking. However, the balance can be difficult and intriguing, particularly when considering conduct deemed unprofessional. Notably, a study published in *J Vasc Surgery* aimed to look into the matter of “unprofessional behavior” by fellow trainee colleagues [85], only to later be retracted from the same journal [86, 87]. The reason for the retraction was among others the inappropriate categorization of other persons’ behavior based on simple views on the said persons’ social media profile (e.g., a photo with a drink in hand; from a beach during vacation, or similar). This also created a response in the journal about who is to judge what is right and wrong [88], as well as a considerable “twitterstorm” (link here: https://twitter.com/JVascSurg/status/1286831352520888320). Indeed, the debate even led to the #MedBikini hashtag used by women doctors and allies globally that were offended by what male colleagues would deem inappropriate or unprofessional attire. Again, this has testified to the important role of SoMe to focus on diversity, gender equity, breaking down biases and stereotypes, and leveling out the playing field—with immediate action taken by journal editors [89].

Hence, several factors need to be kept in mind when interpreting the role and importance of debates and the content a hand. Suggestions to appropriate debate and collegial behavior for SoMe are available and published by societies or specialist journals [19, 90–95] and, importantly also, by most institutions these days. The most overarching point for surgeons and medical professional alike is nonetheless to maintain patient confidentiality and dignity at all times.

Patients may engage with surgeons on SoMe, which can be good thing; patients or their next of kin may learn about conditions in plain English (or the language in question at the platform), it may encourage public engagement, and doctors may get a perspective from the patient on priorities, wishes, thoughts, and needs. However, such contact can also potentially be concerning when crossing boundaries or conducted in an unprofessional manner. Some subspecialties may in particular have a fine line between providing information and bordering on actual advertisements for services [96–99].

Notably, technical issues should not be forgotten. A Twitter handle or social media account may be hacked or security breached. This should be considered both for the individual person but also if unusual or largely unexpected information is distributed from an account with unclear or largely deviating information.

## Conclusions

Social media and their platforms have become yet another tool in the surgeon’s armamentarium for communication and dissemination of research, and for interacting with colleagues and the public. As such, SoMe platforms and several communities have become effective for sharing knowledge and opinions. Some caveats and pitfalls need to be considered and more research is needed into the real-world impact of these platforms. However, the sound use of this dissemination technology has allowed cutting-edge research to reach a wider audience with a lower threshold for discussing new information. Hence, we believe these channels will continue to shape surgeons’ way of obtaining new information, sharing new data, and engaging in scientific debates in the near future.

## Data Availability

Not applicable.
